# Draft Genome Sequence of the Birch Tree Fungal Pathogen Taphrina betulina UCD315

**DOI:** 10.1128/MRA.01255-19

**Published:** 2019-11-27

**Authors:** Padraic Heneghan, Adam P. Ryan, Darragh Nimmo, Claudine Duggan, Paurush Kumar, Peadar O’Gaora, Eoin Ó.'Cinnéide, Kevin P. Byrne, Kenneth H. Wolfe, Caoimhe E. O’Brien, Geraldine Butler

**Affiliations:** a School of Biomedical and Biomolecular Sciences, Conway Institute, University College Dublin, Dublin, Ireland; b School of Medicine, Conway Institute, University College Dublin, Dublin, Ireland; Vanderbilt University

## Abstract

Taphrina betulina is the ascomycete yeast that causes the formation of witches’ brooms in birch trees. Here, we report the first draft genome sequence of *T. betulina*, from strain UCD315, isolated from soil in Ireland. The genome is haploid and 12.5 Mb long.

## ANNOUNCEMENT

*Taphrina* species are plant pathogens in the subphylum Taphrinomycotina of the phylum Ascomycota (1). *Taphrina* species cause plant deformity diseases in a diversity of tree species, including *Prunus* (edible fruit trees and shrubs), *Cerasus* (sour cherry), and *Populus* (poplar) (2). Taphrina betulina was first described in Norway in 1883 (3). It infects Betula pubescens (downy birch), Betula nana (dwarf birch), crosses between the two species (Betula intermedia), and Betula pendula (silver birch) (1, 4). Infection results in host tissue deformities, such as nest-like tumors called “witches’ broom” (5). Ultimately, infection affects the diameter, height, and life span of the tree (4).

*T. betulina* UCD315 was isolated from soil near Lough Corrib, County Galway, Ireland (global positioning system coordinates, 53.4344816, –9.1534624). The yeast was cultured at room temperature on yeast extract-peptone-dextrose (YPD) medium with chloramphenicol (3% [wt/vol]) and ampicillin (10% [wt/vol]). The species was identified by sequencing the internal transcribed spacer (ITS) of the ribosomal DNA (rDNA) gene locus (GenBank accession number MN540705). Genomic DNA was extracted and purified using Qiagen’s QIAamp DNA minikit. Libraries with an insert size of 800 bp were made from genomic DNA and sequenced by BGI Tech Solutions using an Illumina HiSeq 4000 instrument with 150-bp paired-end reads (9.5 million spots). All parameters used for sequence assembly and analysis are available at https://www.doi.org/10.6084/m9.figshare.9775517.

Low-quality reads (1.69 million) were trimmed using Skewer v0.2.2 (6). The genome was assembled from all reads using SPAdes v3.11.1 with the “careful” parameter (7). Based on coverage-versus-length analysis (8), contigs below 72× coverage or 1-kb length were removed. The results were then analyzed using QUAST v4.6.1 (9). The genome size was 12.5 Mb with an *N*_50_ value of 321 kb, an *L*_50_ value of 16, an average coverage of 101×, and a GC content of 49.8%. The largest contig is 682,403 bp. This is similar in size and contiguity to other sequenced *Taphrina* genomes, which range from 11.9 to 15.7 Mb (2). Using BUSCO v3.0.1 (10), genome completeness was estimated at 91.5% (compared to the Ascomycota lineage data set). This is similar to the other eight *Taphrina* genomes (2, 11), which have an average completeness of 91.95% (ranging from 89.6% to 93.1%). The mitochondrial genome was assembled as a 42.9-kb contig (GenBank accession number VWSI01000053).

Analyses using SAMtools v1.1.19 (12), Burrows-Wheeler Aligner MEM (BWA-MEM) v0.7.12-r1039 (13), and Genome Analysis Toolkit (GATK) v4.0.1.2 (14) with default settings identified very small numbers of putative heterozygous single-nucleotide polymorphisms (1,385) and insertion/deletions (181), suggesting that the genome is haploid.

### Data availability.

This whole-genome shotgun project has been deposited in DDBJ/ENA/GenBank under the accession number VWSI00000000 and the raw reads under SRA number SRX6812536. These data are also available under BioProject number PRJNA564291. The ITS sequence is available at accession number MN540705, and the mitochondrial genome is available under accession number VWSI01000053.
